# Rifampicin Attenuated Global Cerebral Ischemia Injury via Activating the Nuclear Factor Erythroid 2-Related Factor Pathway

**DOI:** 10.3389/fncel.2016.00273

**Published:** 2016-11-29

**Authors:** Beibei Chen, Huimin Cao, Lili Chen, Xuemei Yang, Xiaoyan Tian, Rong Li, Oumei Cheng

**Affiliations:** ^1^Department of Neurology, The First Affiliated Hospital, Chongqing Medical UniversityChongqing, China; ^2^Department of Neurology, Jiangjin Central Hospital of ChongqingChongqing, China; ^3^Laboratory Research Center, The First Affiliated Hospital, Chongqing Medical UniversityChongqing, China; ^4^The Second People's Hospital of Banan DistrictChongqing, China; ^5^The Key Laboratory of Biochemistry and Molecular Pharmacology, Department of Pharmacology, Chongqing Medical UniversityChongqing, China

**Keywords:** rifampicin, global cerebral ischemia, delayed neuronal death, nuclear factor erythroid 2-related factor 2, heme oxygenase-1, cyclooxygenase-2

## Abstract

**Background:** Recent studies have found that rifampicin has neuroprotective properties in neurodegenerative diseases. However, the exact mechanisms of action remain unclear. The nuclear factor erythroid 2-related factor 2 (Nrf2) has been considered a potential target for neuroprotection. In this study, we examined whether rifampicin exhibits beneficial effects mediated by the Nrf2 pathway after global cerebral ischemia (GCI).

**Methods:** Rats were randomly assigned to four groups that included a sham group and three treatment groups with global ischemia-reperfusion [control, rifampicin, and rifampicin plus brusatol (an inhibitor of Nrf2)]. Rats were subjected to transient GCI induced by bilateral common carotid artery occlusion for 20 min with systemic hypotension by blood withdrawal. The Morris water maze test was performed for neurobehavioral testing, whereas the pathological changes were investigated using HE and TUNEL staining. The protein expression of Nrf2, hemeoxygenase-1 (HO-1) and cyclooxygenase-2 (COX-2) in the hippocampus were analyzed by Western blotting. The immunofluorescence staining was used to determine the distribution of Nrf2.

**Results:** Rifampicin treatment significantly improved spatial learning ability compared with the control group, which was consistent with the pathological changes. In addition, rifampicin significantly elevated the nuclear expression of Nrf2, Nrf2 downstream anti-oxidant protein, HO-1 compared with the control group, and it simultaneously downregulated the expression of COX-2 in the hippocampus on day 3 after ischemia-reperfusion. Interestingly, the forenamed effects of rifampicin were abolished by pretreatment with brusatol, a specific inhibitor of Nrf2 activation.

**Conclusions:** Rifampicin exerts neuroprotective effects against global cerebral ischemia, which may be attributed to activation of the Nrf2 pathway.

## Introduction

Global cerebral ischemia (GCI) is a clinical outcome that can occur as a consequence of conditions including cardiac arrest, severe dysrhythmias, reversible severe hypotension (Liang et al., 2008). Transient GCI is a cerebrovascular condition that diminishes the cerebral blood flow by up to 10% (Fisher and Bogousslavsky, 2000) and usually leads to selective delayed neuronal death (DND) of pyramidal neurons in the hippocampus, especially in the cornu ammonis area 1 (CA1) in experimental animal models (Pulsinelli and Brierley, 1979; Kirino, 1982). The complicated mechanisms of DND have been extensively studied. One of the critical pathophysiological features of GCI is oxidative stress induced by ischemia/reperfusion (I/R) injury (Liang et al., 2008). Thus, reducing oxidative stress may be one of the logical approaches for inhibiting DND and alleviating GCI.

Nuclear factor erythroid 2-related factor 2 (Nrf2) is considered an important cytoprotective regulator against oxidative stress (Kensler et al., 2007). During oxidative stress, Nrf2 is released from Kelch-like ECH-associated protein 1 (Keap1) in the cytoplasm, translocates into the nucleus and up-regulates expression of many antioxidant and detoxification genes, including HO-1, glutathione S-transferases (GSTs), and NAD(P)H quinone oxidoreductase (Yang et al., 2015). Recent evidence has proven that activation of Nrf2 can attenuate oxidative injury induced by focal cerebral ischemia, which is equivalent to translocation from the cytoplasm to the nucleus, and promotes expression of a variety of antioxidant genes (Chen et al., 2012; Li et al., 2013). Among these antioxidant genes, HO-1 has been implicated to be particularly important in neuroprotection against cerebral ischemia, as evidenced by HO-1 knockout mice exhibiting greater ischemic damage compared to wild type mice (Kim et al., 2011). In addition, the HO-1 promoter is known to have a large number of ARE sequences to which Nrf2 can bind to induce its expression in a preferential manner (Kensler et al., 2007). Consequently, the Nrf2/HO-1 pathway might be considered a potential target against oxidative stress in GCI.

Rifampicin is a member of a class of broad-spectrum antibiotics that are fermentation products of Nocardia mediterranea and is widely used against mycobacterium tuberculosis (Blanchard, 1998; Yulug et al., 2004). It is formed from napthohydroquinone or naphthoquinone chromophores spanned by an aliphatic ansa chain (Tomiyama et al., 1996). Recent findings have shown that rifampicin reduced apoptosis in focal ischemic stroke and increased the survival of nigrostriatal dopaminergic neurons in models of Parkinson's disease (Yulug et al., 2004; Oida et al., 2006; Chen et al., 2010). However, the exact mechanisms of action remain unclear. It has not been reported whether rifampicin inhibits DND after GCI. In the present study, we hypothesized that rifampicin may decrease DND by improving the ischemic tolerance of neurons by promoting the activation of Nrf2 in the hippocampus CA1 after GCI.

We have previously shown that COX-2 is a key enzyme resulting in neuronal apoptosis in GCI (Cheng et al., 2010, 2011). Nrf2 is also involved in reducing the expression of inflammation-related factors, such as inducible nitric oxide synthase (iNOS), COX-2 and IL-6, in LPS-stimulated macrophages and endotoxin-shocked mice (Mo et al., 2014). Therefore, in the present study, we investigated whether rifampicin could reduce expression of COX-2 by activating Nrf2.

Our overall aim was to investigate whether rifampicin has neuroprotective effects in the hippocampus of rats with GCI. Furthermore, we also explored whether the protection provided by rifampicin would occur through activation of the Nrf2/HO-1 pathway and reduce the expression of COX-2.

## Materials and methods

### Animals and groups

Adult male Sprague Dawley rats weighing 250–300 g provided by the Experimental Animal Center of Chong Qing Medical University were used. All animal experiments were performed in accordance with the guidelines for the care and use of laboratory animals and the international guidelines for the ethical use of laboratory animals. The study was approved by the Institutional Animal Care and Use Committee at Chong Qing Medical University. All efforts were made to minimize animal suffering, reduce the number of animals used and utilize alternatives to *in vivo* techniques, if possible. Rats were acclimatized for 7 days before the experiments, had free access to food and tap water and were maintained at 21 ± 2°C under a 12 h dark and light cycle.

Seventy two adult male SD rats were randomly assigned to four groups that included a sham group and three treatment groups with global ischemia [control (I/R group), rifampicin (RFP group), and rifampicin plus brusatol (RFP + Bru, an inhibitor of Nrf2)], 18 rats in each group. They were tested behaviorally (*n* = 6), histopathology (*n* = 4), and molecular biology (*n* = 4). I/R group underwent the GCI operation. RFP group received the GCI operation and were also treated with rifampicin. Rats in the RFP + Bru group received the GCI operation and were also treated with rifampicin plus brusatol.

### Drug treatment

Rifampicin (purchased from Huapont Pharm. Co., Chongqing, China) was dissolved in saline used as a vehicle and diluted to 2 mg/ml. After 30 min of reperfusion, rats received an intraperitoneal injection of rifampicin (20 mg/kg) or vehicle for 7 days. All rats received left lateral ventricle puncture (anteroposterior, −1.1 mm; lateral, 1.5 mm; depth, 4.5 mm; from bregma), in RFP + Bru group, 30 min prior to ischemia animals received Nrf2 inhibitor brusatol by intracerebroventricular infusion (1 mg/kg, dissolved in 5 μl 1% DMSO). Sham-operated group also received equal volume vehicle injection after surgery.

### Establishment of transient global ischemic model

The animals were fasted for 12 h, but they were allowed free access to water before surgery. Transient global ischemia was induced in SD rats via the 2-vessel occlusion (2-VO) model. The model and the criteria for model success and exclusion were previously described (Cheng et al., 2012). Briefly, after injection of 4% chloral hydrate (400 mg/kg, i.p.), animals were placed on a thermostat plate, and their body temperature was maintained close to 37°C. Both common carotid arteries were exposed and carefully separated from the vagus nerve and surrounding tissues, and the right jugular vein was cannulated. Blood (2.5 ml/100 g) was withdrawn via the jugular vein into a warmed heparinized syringe, and then both carotid arteries were temporary occluded with micro-aneurysm clips for 20 min. After releasing the clamps, the extracted blood was slowly reinfused and the incisions were sutured. Sham-operated animals, which served as controls, were subjected to the same surgical procedures but without occlusion of the carotid arteries and induction of hypotension.

At 3 or 7 days after reperfusion, the animals were deeply anesthetized and transcardially perfused with saline followed by 4% paraformaldehyde. The brains were removed and postfixed in 4% paraformaldehyde for at least 24 h at 37°C. The slices at day 7 after reperfusion were embedded in paraffin, cut into coronal serial sections (−3.3 to −4.5 mm from Bregma) at a thickness of 4 μm, and prepared for TUNEL and HE staining. The slices at day 3 after reperfusion were dehydrated and cryoprotected in gradient sucrose; frozen coronal sections (10 μm) were made on a Leica sliding microtome and were cut from Bregma −3.3 to −4.5 mm and used to perform immunofluorescence assays as previously described (Cheng et al., 2010).

### Morris water maze

Hippocampus-dependent spatial learning and memory abilities were evaluated with the Morris water maze (MWM) test. Briefly, the water maze (150-cm diameter) was divided into four quadrants. A hidden platform (12-cm diameter) was placed 2 cm below the water surface in the center of one quadrant during pretraining and testing. The rats were subjected to pretraining (the navigation test) on postoperative days 7–12 twice daily, in the morning and afternoon, which was followed by testing (the orientation test) without the platform.

During the pretraining, rats were released into the maze from a randomly selected quadrant and with all animals using the same order. The escape latency to find the underwater platform was recorded. If the rat failed to reach the hidden platform within 60 s, then it was manually guided to the platform, where it was placed for 15 s. Each animal performed four training sessions from different starting quadrants per day. All animals had been pre-trained for 4 consecutive days before the surgery. The escape latency (seconds) and swimming speed (cm per second) were analyzed. Data from trials in each daily session were averaged for each rat.

To assess spatial learning ability, the platform was removed from the pool, and the animals were subjected to a 60 s probe trial following the last training session to fond the original platform (target crossings). The proportion of time spent in the target quadrant, the number of platform crossings, and the swimming speed (cm per second) were monitored and recorded by a video camera linked to a computer-based image analyzer.

### Hematoxylin and eosin (HE) staining

HE staining was performed to determine the neuronal loss in both hippocampi of each animal. For HE staining, sections were deparaffinized, hydrated, stained with hematoxylin for 3 min, differentiated by 1% hydrochloric acid alcohol for 1 s, and stained with eosin for 3 s. Stained sections were dehydrated, mounted with neutral balsam, and covered with a coverslip. The number of CA1 pyramidal cells was counted at 400x magnification. Only cells with an obvious nucleus and nucleolus were included. Images were taken by light microscope (Olympus).

### *In-situ* detection of DNA fragmentation (TUNEL staining)

For the analysis of neuronal apoptosis, TdT-mediated deoxy-uridine triphosphate nick end labeling (TUNEL) staining was used according to the manufacturer's protocol of TUNEL POD (Cat.No.11718096001, Roche, Germany). After being deparaffinized with xylene and hydrated in decreasing concentrations of alcohol solutions, the sections were treated with 3% H2O2 diluted by methanol for 15 min, digested by proteinase K solution for 60 min at 37°C, treated with Triton-X100 transparent (0.01%) for 20 min, added with TUNEL reaction mixture (50 ml) overnight at 4°C, incubated with POD for 60 min at 37°C, and the POD of the sections was demonstrated with DAB substrate kit (Lot: ZLI-9017, Zhongshan). To reveal the nuclear morphology, sections were further stained with hematoxylin. Finally, sections were mounted with coverslips. Five slices were taken from each rat brain. We randomly chose four non-overlapping view-fields from hippocampal CA1 regions of both hemispheres from each animal. The total number of apoptotic cells was counted by an investigator who was blinded to the experimental conditions and who performed all assessments at 400x magnification.

### Double immunofluorescence staining

After being washed in PBS, coronal sections were pretreated with 0.3% Triton X-100 for 20 min at 37°C and then incubated with 3% bovine serum albumin for 30 min at 37°C. Then, the sections were incubated overnight at 4°C with rabbit anti-Nrf2 (1:100, Abcam) and mouse anti-MAP2 (microtubule associated protein 2) antibody (1:50, Santa Cruz) to label neurons. These antibodies were detected using the appropriate fluorescence-labeled secondary antibodies at 1:100 (goat anti-mouse conjugated to DyLight 488 or goat anti-rabbit conjugated to DyLight 549, Abbkine). DAPI (1 ng/μl) was used for incubation for nucleus visualization. Finally, the sections were coverslipped and analyzed by immunofluorescent confocal microscopy (Leica, Germany).

### Western blot analysis

The animals were euthanized under general anesthesia on day 3 after surgery, and total protein, nuclear protein and cytoplasmic protein were extracted from both hippocampus as described previously (Cheng et al., 2010). Western blotting procedures followed standard protocols (Shimamura et al., 2006). Equal amounts (30 μg) of total protein and nuclear protein were separated in 10% SDS-polyacrylamide gel and transferred to PVDF membranes. Primary antibodies included anti-β-actin (1:1000, Boster), anti-Histone H3 (1:1000, Millipore), anti-Nrf2 (1:1000, Abcam), anti-HO-1 (1:1000, Abcam), and anti-COX-2 antibody (1:1000, Abcam). Finally, protein bands were detected using an enhanced chemiluminescence (ECL) kit (ThermoFisher Scientific) according to the manufacturer's protocol. The IOD of each band was measured using a gel-image analyzing system (Fusion Optix, USA). β-actin was used as an internal control to normalize the target protein expression.

### Statistical analysis

**Figure 1:**
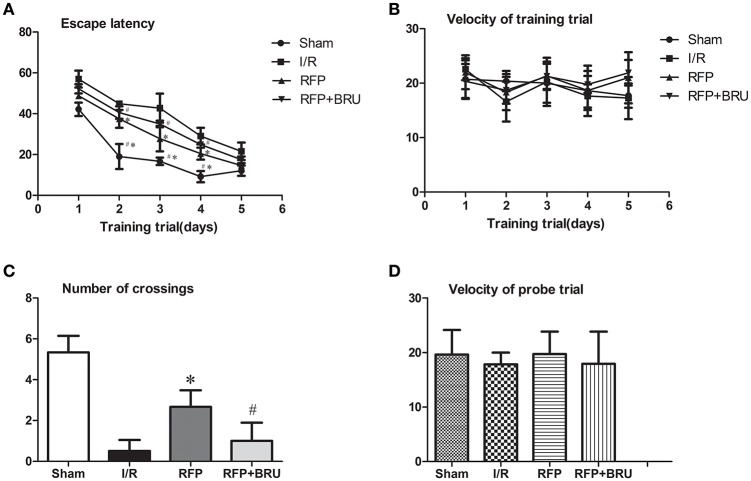
**Escape latency for the training trials (A)** and velocity **(B)** during the place navigation stage. Number of crossings **(C)** and velocity **(D)** in the probe trial. The data are expressed as the mean ± *SD* (*n* = 6 in each group). ^*^p < 0.05 compared with I/R group, #*p* < 0.05 compared with RFP group.

Statistical analyses were performed using SPSS 19.0 (SPSS Inc., Chicago, IL, USA). All data are presented as the mean ± SD (standard deviation). All data were performed by an investigator who was blinded to the experimental conditions. Data recorded in the navigation test (swimming velocity, escape latency, Figure 1) were analyzed by two-way ANOVA with repeated measures, followed by a LSD test for *post-hoc* comparisons to compare differences between groups. The remaining data were evaluated by one-way ANOVA, followed by the Student-Newman–Keuls test for post hoc comparisons to compare differences between groups (statistical significance, *P* < 0.05).

## Results

### Morris water maze test

#### The navigation test

The swimming velocity and mean latency for finding the platform (escape latency) during the place navigation test are shown in Figure 1. In all groups, the escape latencies showed a progressive reduction over successive trials, especially in the sham-operated and rifampicin-treated rats. The I/R group spent a longer time searching for the hidden platform compared to the sham group. The RFP group exhibited shorter exploration times to find the hidden platform location compared to the I/R group, and brusatol treatment prior to ischemia resulted in a significantly longer escape latency for locating the submerged platform during the first four trials, relative to rats that received rifampicin treatment (Figure 1A, *P* < 0.05). It was observed that all animals in all three groups could swim easily and with similar swimming velocities at all time points (Figure 1B, *P* > 0.05).

#### The orientation test

The swimming velocity and number of crossings in the orientation test are shown in Figure 1. The orientation test was conducted by removing the platform and allowing the rat to swim for 60 s to search for the platform. The number of crossings over the platform location during the orientation test was lower in the I/R group compared with Sham group, whereas brusatol pretreated rats demonstrated a poorer performance with Nrf2 inhibitor pretreatment versus rats receiving rifampicin monotherapy (Figure 1C, *P* < 0.05). The swimming velocity did not differ significantly (Figure 1D, *P* > 0.05) among the three groups in this trial.

### Hematoxylin and eosin (HE) staining

**Figure 2:**
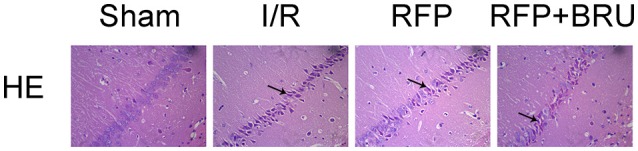
**Effects of rifampicin on ischemia-induced histological changes in hippocampal CA1 neurons 7 days after ischemia (×200, HE staining)**. Abnormal cells are indicated by the arrows.

HE histology revealed characteristics of delayed cell death at day 7. There was no significant neuronal damage or neuronal loss in the hippocampal CA1 subregion in the sham group. Pyramidal neurons were arranged in 2–3 cell layers, and in individual neurons the nucleus was full, nucleolus was clear, and cell outlines were clear (Figure 2). In the I/R group, the neurons were sparsely arranged with fuzzy cell outlines, which revealed marked neuronal damage, shrunken cell bodies, vacuolation and pyknotic nuclei. The number of cells in the RFP group with an eumorphism was larger than that in the I/R group. The pathology was similar between rats pretreated with brusatol and those in the I/R group.

### TUNEL staining

**Figure 3:**
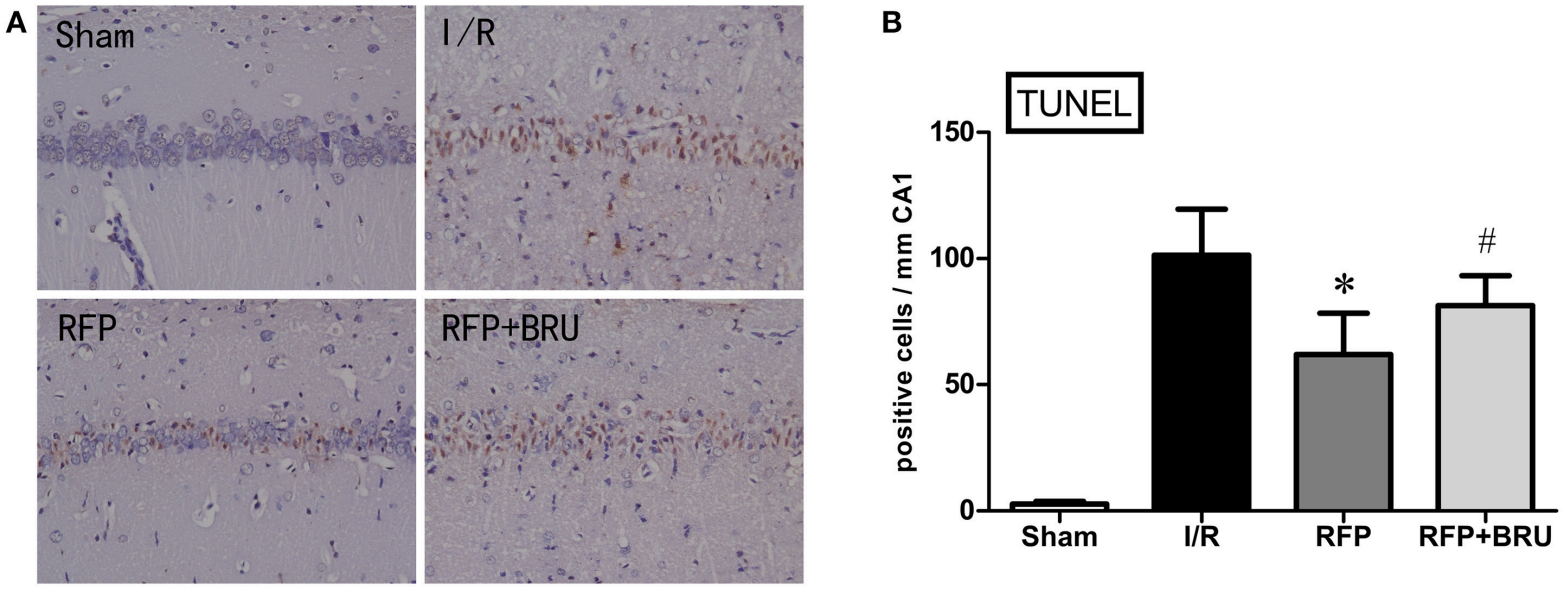
**(A)** Effects of rifampicin (RFP) on the number of TUNEL-positive cells in the hippocampal CA1 region 7 days after ischemia (×200, TUNEL staining). **(B)** Quantification was performed by counting the number of apoptotic-like neurons in the medial CA1 pyramidal cell layer (mean ± *SD, n* = 4 in each group). Apoptotic-like neurons were shrunken cell bodies, stained brown (indicated by arrows). ^*^p < 0.05 compared with I/R group, #*p* < 0.05 compared with RFP group.

There were few TUNEL-positive cells in the sham group, whereas a large number of TUNEL-positive neurons were observed in the CA1 region of rats in the I/R group at day 7 after global ischemia (Figure 3A). In contrast, the number of apoptotic cells was significantly lower in the RFP group than in the I/R and RFP+Bru groups (Figure 3B, *p* < 0.05).

### Expression of Nrf2 in the hippocampus

**Figure 4:**
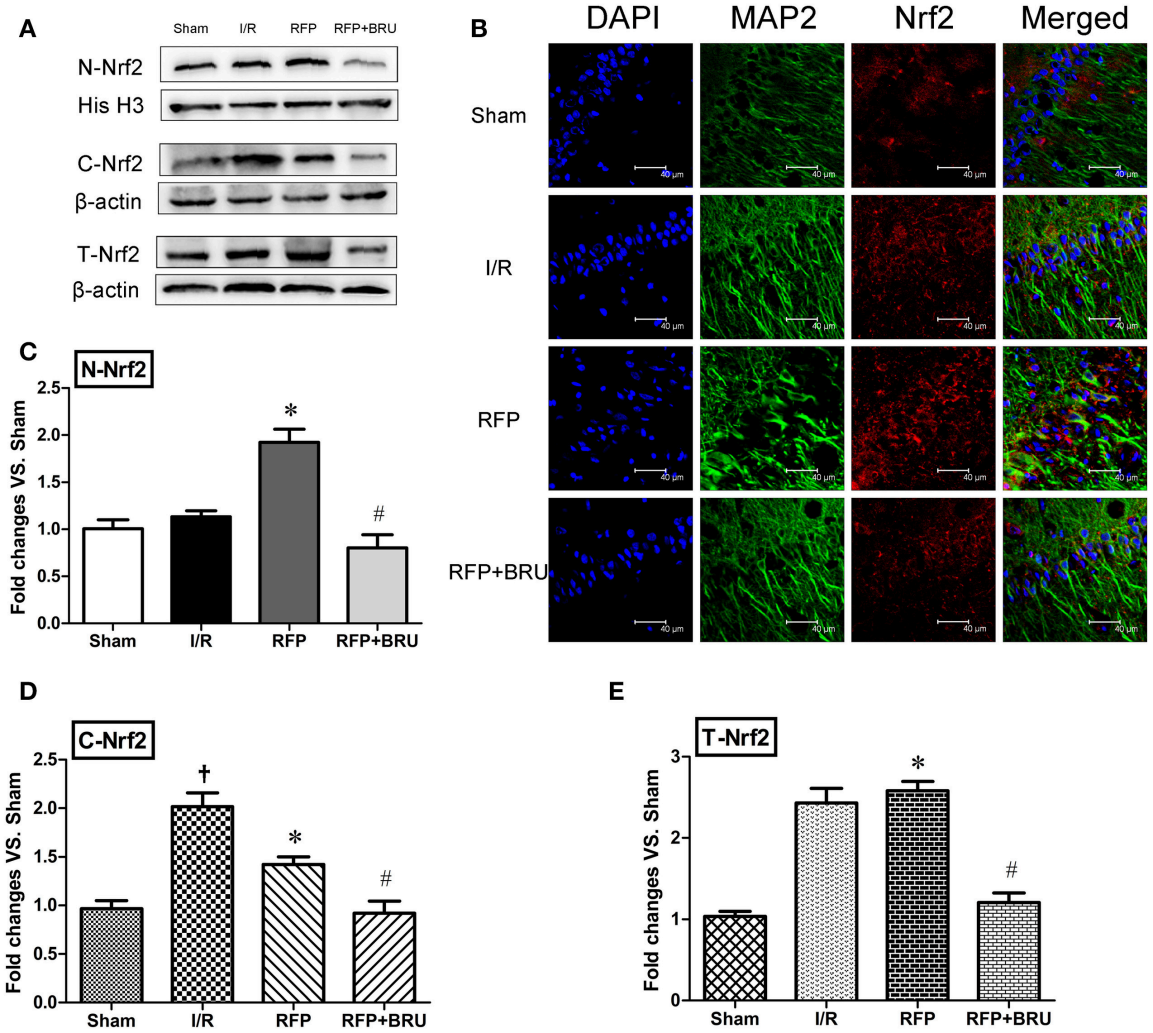
**Effects of rifampicin (RFP) on Nrf2 protein distribution**. Representative blot of Nrf2 protein obtained from cytoplasmic, nuclear, and total extracts. **(A)** Effects of rifampicin on Nrf2 protein distribution. **(B)** Immunofluorescence staining was performed for Nrf2 and MAP2 in hippocampal CA1 region 3 days after reperfusion (magnification, 800×), scale = 40 μm. Data analyses for Nrf2 protein from the nuclear **(C)**, cytoplasmic **(D)**, and total extracts **(E)** expressed as the fold change vs. the sham group (mean ± *SD, n* = 4 in each group). ^†^*p* < 0.05 compared with sham group, ^*^p < 0.05 compared with I/R group, #*p* < 0.05 compared with RFP group.

The band of Nrf2 protein expression (nucleus protein, cytoplasm protein and total protein) was shown in Figure 4A. The cytoplasm protein levels of Nrf2 in the hippocampal CA1 neurons of I/R group rats were up-regulated compared with those of the sham group (Figure 4D, *p* < 0.05). However, the nucleus protein Nrf2 was not obviously increased compared with the sham group (Figure 4C, *p* < 0.05). Rifampicin treatment significantly increased the nucleus protein levels of Nrf2 (Figure 4C, *p* < 0.05) and decreased the cytoplasm protein levels of Nrf2 (Figure 4D, *p* < 0.05) compared with the I/R group. Total protein levels of Nrf2 was shown in Figure 4E. Compared to the sham group, there were no distinct differences in the cytoplasm protein expression of Nrf2, but the nuclear expression was significantly enhanced after rifampicin treatment. Interestingly, brusatol pre-treatment markedly attenuated the nuclear translocation of Nrf2 3 days after reperfusion. Further, the representative images by confocal analysis of double immunohistochemistry for Nrf2 and MAP2 revealed a higher nuclear immunofluorescence in hippocampal CA1 neurons in the RFP group compared to the I/R and RFP+Bru groups (Figure 4B).

### Expression of HO-1 in the hippocampus

**Figure 5:**
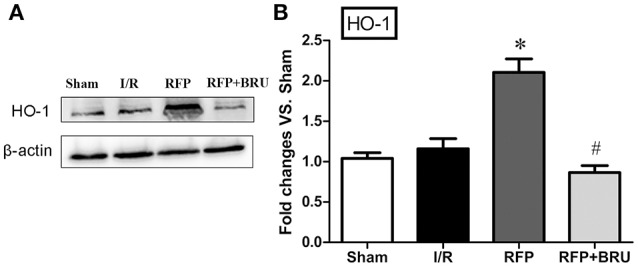
**Effect of rifampicin on HO-1 levels in the hippocampus (mean ± ***SD***, ***n*** = 4). (A)** Total protein in sham, vehicle, rifampicin, and rifampicin+brusatol pre-treatment animals were subjected to Western blotting for HO-1 and β-actin expression. **(B)** Blotting bands were scanned, and the data are expressed as the fold change vs. the sham group. ^*^p < 0.05 compared with I/R group, #*p* < 0.05 compared with RFP group.

In the I/R group, we observed that protein level of HO-1 that was no significant difference compared to the basal levels in the sham group 3 days after reperfusion. Western blot analysis showed that, consistent with the DNA binding activity of Nrf2, HO-1 levels were significantly increased by rifampicin compared with the I/R group 3 d after reperfusion. Brusatol attenuated this increase (Figure 5).

### Expression of COX-2 in the hippocampus

**Figure 6:**
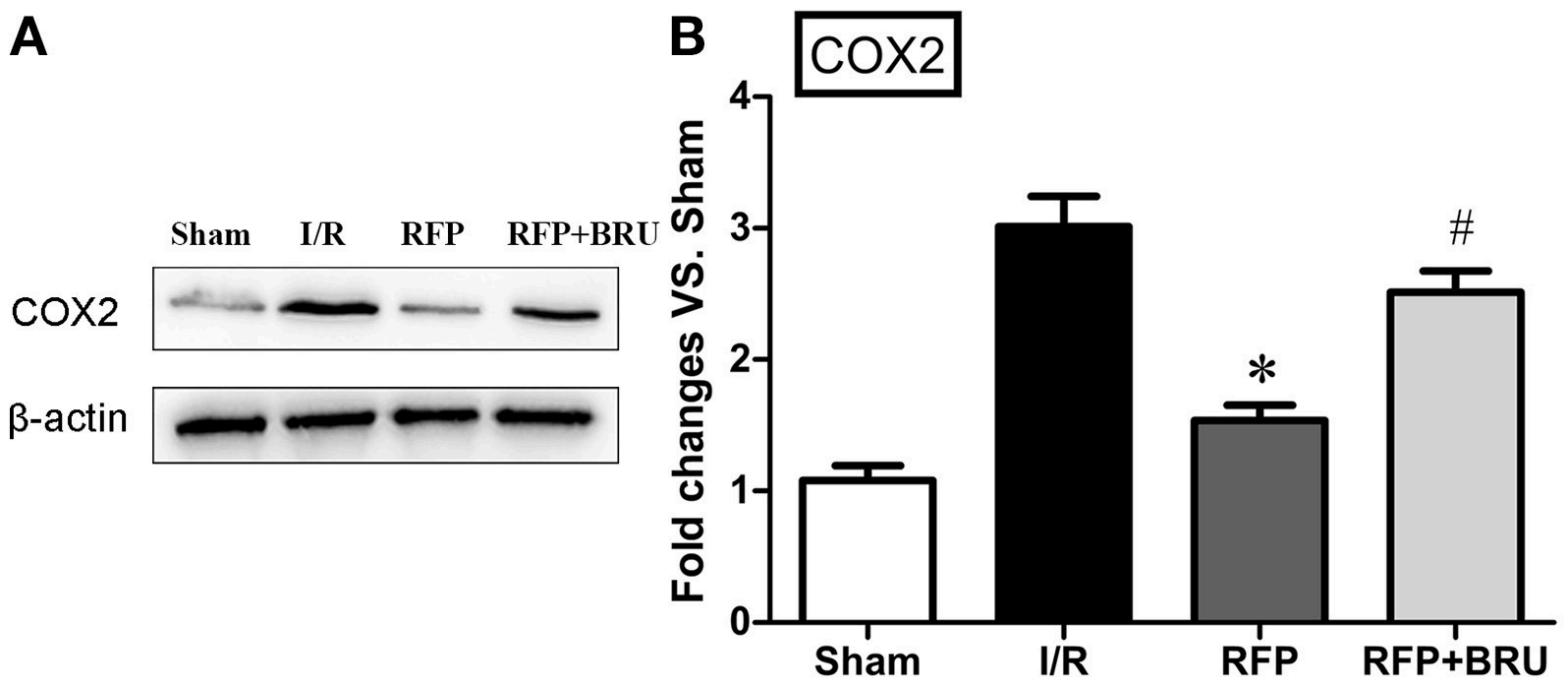
**Effect of rifampicin on COX-2 levels in the hippocampus (mean ± ***SD***, ***n*** = 4)**. Representative Western blot and Western blot quantitation normalized relative to β-actin. ^*^p < 0.05 compared with I/R group, #*p* < 0.05 compared with RFP group.

In our previous study, GCI was shown to cause marked elevation in COX-2 expression, which is responsible for neuronal injury or death (Cheng et al., 2011). The levels of COX-2 were determined in brain tissue using Western blot analysis. Western blotting for COX-2 revealed that ischemic surgery led to a significant increase in COX-2 levels in the I/R group relative to sham group (Figure 6, *p* < 0.05). The treatment with rifampicin after surgery led to a notable reduction in COX-2 content compared with the control group, whereas brusatol attenuated the decrease (Figure 6, *p* < 0.05).

## Discussion

In the present study, we found several important findings. First, rifampicin decreased the DND of pyramidal neurons and improved cognitive impairments resulting from GCI. Second, rifampicin also promoted nuclear translocation of Nrf2 in the CA1 region of the hippocampus and increased the expression of HO-1. Third, the present study demonstrated that rifampicin significantly inhibited the expression of COX-2 in the hippocampus following GCI. Finally, all of these effects were reversed by administration of brusatol, an inhibitor of Nrf2.

DND in the brain has been considered a central pathological change in GCI. In rats subjected to GCI, it is accepted that the cortex and hippocampus are the most vulnerable regions, and GCI usually leads to selective DND of pyramidal neurons, particularly in the CA1 region, which is associated with cognitive dysfunction and disruption of spatial learning and memory in rats (Cheng et al., 2012).

Although, evidence suggests that rifampicin ameliorates brain damage in focal cerebral ischemia (Yulug et al., 2004), treatment with rifampicin in GCI has not yet been investigated. Our results showed that ischemic rats spent a longer time searching for the hidden platform during the navigation test and showed fewer crossings over the platform area in the orientation test. However, the average swimming velocity was similar among the groups in both the place navigation stage and probe trials, which reveals that motor function was unaffected after GCI. After administration of rifampicin, the performances in the MWM test were significantly improved. This suggested that treatment with rifampicin significantly ameliorated GCI/R-induced cognitive dysfunction.

DND was mainly evidenced by apoptosis observed at 3–4 days that peaked 7 days after GCI (Kirino et al., 1984; Kihara et al., 1994; Nitatori et al., 1995). In this study, we found that pathologic changes, including neuronal cell loss, smaller sizes, irregular shapes, deepened cytoplasmic staining, and nuclei shrinkage after ischemia, the pyramidal cells were ranged in order in sham group as revealed by HE staining. After rifampicin treatment, a significantly reduced number of TUNEL-positive cells and increased number of surviving neurons was observed in the hippocampus CA1 at 7 days compared with the I/R group. Our experiments confirmed that rifampicin-related improvement in cognitive functions after GCI was associated with reduced cell apoptosis and increased survival of hippocampal CA1 neurons.

**Figure 7:**
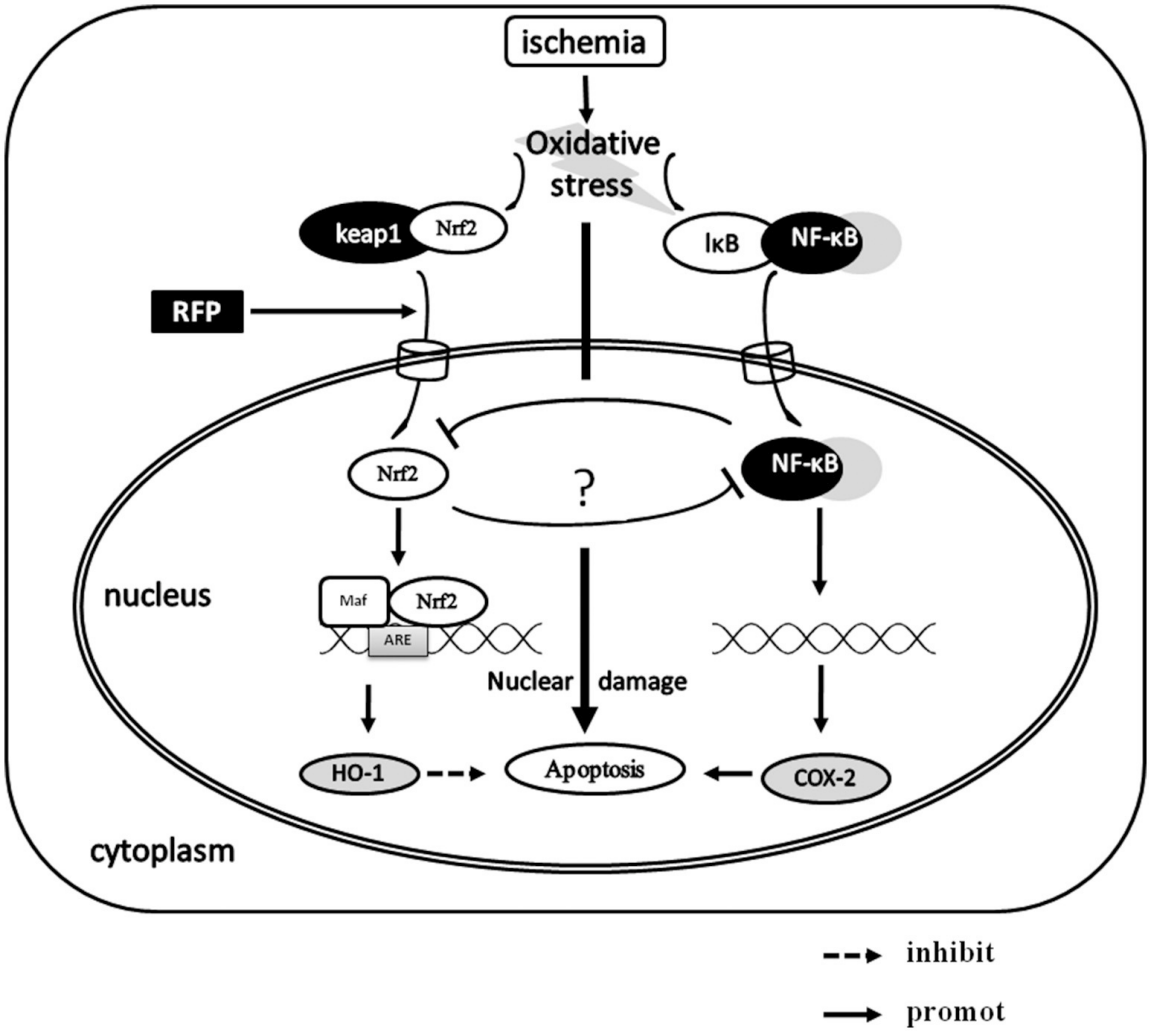
**The possible neuroprotective mechanism of RFP in GCI**. The neuron cells are faced with an insult such as oxidative stress after GCI, Nrf2 activation is an important endogenous antioxidant mechanism that protects against oxidative stress. RFP significantly elevates Nrf2 levels in the nucleus, then Nrf2 binds to the ARE and simultaneously promoting the expression of HO-1. In addition, the elevating of nuclear Nrf2 could inhibit the expression of COX-2, which may be mediated by NF-κB. As the result, the cell apoptosis was reduced.

Oxidative injury was considered the main pathological mechanism of DND (Chan et al., 1998). Nrf2 activation is an important endogenous antioxidant mechanism that protects against oxidative stress (Guo et al., 2015). During ischemia, the expression level of Nrf2 is enhanced, and it is partly released from Keap1 and translocated to the nucleus, where it triggers the expression of several dozen cytoprotective proteins, such as HO-1 and NAD(P)H (Saleem et al., 2008; Tulsulkar and Shah, 2013). More importantly, that study found that promoting the activation of Nrf2 has neuroprotective effects (Ding et al., 2014). In this study, our data showed that, compared with the sham group, the cytoplasm protein levels of Nrf2 in the I/R group were up-regulated, whereas the Nrf2 protein was not obviously increased in the nucleus. This demonstrated that endogenous upregulation of Nrf2 was not sufficient to protect neurons against GCI injury. Rifampicin significantly elevated Nrf2 levels in the nucleus and simultaneously decreased them in the cytoplasm. The double immunofluorescence for Nrf2 and MAP2 also showed a higher nuclear immunofluorescence in hippocampal CA1 neurons of in the RFP group compared to I/R group. HO-1, a target antioxidant enzyme of Nrf2, is critical for the protection against ischemia and reperfusion (I/R) injury in the brain, Parkinson's disease, and other neurological disorders (Calkins et al., 2009). Our data presented here showed that rifampicin significantly improved the protein levels of HO-1 on day 3 post-ischemia. However, when the Nrf2 inhibitor brusatol was added, the effects of rifampicin on HO-1 protein expression were partially abolished. These findings indicate that rifampicin-induced neuroprotective effects are indeed responsible for the activation of the Nrf2/HO-1 pathway. In addition, the rifampicin treatment plus Nrf2 inhibitor group still showed a neuroprotective effect compared with the I/R group. This finding suggests that in addition to the Nrf2/HO-1 pathway, other mechanisms may be involved in rifampicin-induced neuroprotection. Additionally, this dose of brusatol may incompletely block Nrf2 in GCI. Previous studies confirmed that rifampicin suppressed oxidative stress in rotenone-induced cell death of PC12 cells, and this ability may be attributed to its naphthohydroquinone ring (Chen et al., 2010). In models of Parkinson's disease, rifampicin could also ameliorate oxidative stress, but the effect of rifampicin on the Nrf2/HO-1 pathway was not discussed (Oida et al., 2006; Chen et al., 2010). In this study, we confirmed that rifampicin increased ischemic tolerance of CA1 neurons, which was associated with activation of the Nrf2/HO1 pathway (Figure 7).

Accumulating evidence suggests that the enhanced COX-2 involvement is involved in neuronal death during GCI (Nakayama et al., 1998; Sasaki et al., 2004; Xiang et al., 2007). Our previous studies also demonstrated that COX-2 promoted neuronal apoptosis through caspase 3-dependent apoptosis after GCI (Cheng et al., 2011). To confirm the relationship between nuclear translocation of Nrf2 and neuronal apoptosis, we measured the expression of COX-2 in the hippocampus. We found that rifampicin-treated rats had higher nuclear Nrf2 expression that was accompanied by lower COX-2 expression in the hippocampus compared with the I/R group. However, brusatol, an inhibitor of Nrf2, could restore the expression of COX-2. These results suggested that downregulation of COX-2 may be linked to activation of Nrf2. The finding that an agonist of Nrf2 downregulated the expression of COX-2 was reported in a previous study, where it occurred through inhibition of nuclear factor kappaB (NF-kappaB) activation *in vivo* and *in vitro* of colitis (Lee et al., 2007). Healy and colleagues reported that activation of Nrf2 can directly interfere with the c-Jun N-terminal kinase 2 signaling activity subsequent to negative regulation of the expression of COX-2 *in vitro* in arthritis (Healy et al., 2005). Therefore, consistent with these results, our data demonstrated that COX-2 may be an important downstream target of Nrf2 (Figure 7). However, further studies are required to examine the downregulation of Nrf2 and to determinate the link between Nrf2 and expression of COX-2 *in vitro*.

In conclusion, the present study demonstrates that rifampicin can elicit its neuroprotective effect after GCI/R. The underlying mechanisms may include activation of the Nrf2/HO-1 pathway, which was in part blocked by brusatol. Meanwhile, expression of COX-2 was also inhibited. We demonstrated the unexplored potential of rifampicin for the treatment of GCI/R injury and that pharmacological activation of the Nrf2 pathway may provide neuroprotection.

The gender dependent significant differences in the cell death pathways following ischemic insults and in pharmacotherapy and some diseases have reported (Mathur et al., 2015; Rosano et al., 2015). The limitations of this study is that only male rats were used in present study, so biological sex difference of rifampicin was not investigated.

## Author contributions

BC: Manuscript Preparation, Research project Conceived, and designed the experiments; HC: Manuscript Preparation, Research project; BC, HC: Co-first author; LC, XY, XT, RL: Research project; OC: Corresponding author, Manuscript Preparation, Research project PI.

### Conflict of interest statement

The authors declare that the research was conducted in the absence of any commercial or financial relationships that could be construed as a potential conflict of interest.

## Abbreviations

Nrf2: Nuclear factor erythroid 2-related factor
GCI: Global cerebral ischemia
RFP: rifampicin
HO-1: Hemeoxygenase-1
COX-2: Cyclooxygenase-2
DND: Delayed neuronal death.
